# Nonpathogenic SIV Infection in Sooty Mangabeys Is Characterized by a Lack of Activation and Expansion of Monocytes

**DOI:** 10.1111/jmp.70104

**Published:** 2026-08-13

**Authors:** Caroline Soulas, Hai Duc Nguyen, Summer Siddiqui, Natalie Stahr, Tricia H. Burdo, Francis J. Novembre, Woong‐Ki Kim, Kenneth C. Williams

**Affiliations:** ^1^ Biology Department Boston College Chestnut Hill Massachusetts USA; ^2^ Innate Pharma Marseille France; ^3^ Division of Microbiology Tulane National Biomedical Research Center, Tulane University Covington Louisiana USA; ^4^ Department of Medicine Rutgers Institute of Translational Medicine and Science, Rutgers Biomedical Health and Sciences, Rutgers University New Brunswick New Jersey USA; ^5^ Divsion of Microbiology and Immunology Emory National Primate Research Center, Emory University Atlanta Georgia USA; ^6^ Office of Environmental Health & Safety Florida Atlantic University Boca Raton Florida USA; ^7^ Department of Microbiology & Immunology Tulane University School of Medicine New Orleans Louisiana USA

**Keywords:** CCR8, CD11b, CD14, CD16, CD163, sCD163, SIV

## Abstract

**Introduction:**

Natural hosts of simian immunodeficiency virus (SIV), including sooty mangabeys (SMs), maintain high viremia without progressing to AIDS and AIDS‐associated histopathogenesis. The mechanisms underlying this protection and lack of pathogenesis remain incompletely understood, particularly in relation to monocyte/macrophage activation.

**Methods:**

Plasma soluble CD163 (sCD163), monocyte CD163 expression, and monocyte subset phenotypes were quantified in uninfected and naturally SIV‐infected SMs and compared with uninfected and SIV‐infected rhesus macaques (RMs). Flow cytometry and multivariate analyses were used to assess monocyte activation and subset distribution.

**Results:**

sCD163 levels and CD163 surface expression were increased in SIV‐infected RMs but not SMs. SIV‐infected SMs exhibited reduced CD14^+^CD16^+^ monocyte expansion and lower expression of CD64, MAC387, CD11b, and CCR2 compared with SIV‐infected RMs. Machine learning identified CD14^+^CD16^+^CCR8^+^ monocytes as the top discriminator of SIV infection in SMs.

**Conclusion:**

These findings indicate a lack of monocyte activation in SMs that correlates with non‐pathogenic infection.

## 1 Introduction

Despite high viremia, natural hosts of simian immunodeficiency virus (SIV), including sooty mangabeys (SMs) and African green monkeys (AGMs), rarely develop AIDS and are thus considered “non‐pathogenic hosts” [1]. Similar to human immunodeficiency virus (HIV)‐infected humans and SIV‐infected rhesus macaques (RMs) that are pathogenic hosts of the virus, SMs have high plasma virus, and CD4 T lymphocytes are preferential targets of infection [2, 3]. As with humans and RMs, infected cells in SMs have short half‐lives, mucosal CD4 memory T cells are severely depleted, and immune activation during the acute phase of infection is comparable [4, 5]. Consistent with the absence of chronic immune activation, natural SIV hosts also fail to exhibit increased lymphocyte turnover during infection. Measurements of Ki‐67 expression demonstrate that CD4 and CD8 T cell proliferation remains unchanged in SIV‐infected sooty mangabeys compared to uninfected controls, whereas pathogenic infection in RMs is associated with significantly elevated lymphocyte proliferation [6]. These findings support the notion that tightly regulated immune activation, rather than viral replication per se, is a key determinant of disease progression. Recent genomic and evolutionary studies also provide insight into AIDS resistance in natural SIV hosts. Sequencing of the SM genome has revealed species‐specific divergence in immune regulatory genes, including a C‐terminal TLR4 associated with a blunted response to lipopolysaccharide, and structural variation in ICAM‐2 leading to reduced surface expression, suggesting intrinsic mechanisms that limit innate immune activation [7]. In parallel, cross‐species transmission models show that the SM strain SIVsmE041 can adapt toward increased pathogenicity in humanized systems, with serial passaging resulting in higher viral replication, progressive CD4 T cell decline, and accumulation of nonsynonymous mutations—particularly in *nef* and *env* [8]. Together, these findings highlight the interplay between host genetic adaptations and viral evolution in shaping disease outcomes.

Chronic immune activation is persistent in humans and RMs, but it is transient and rapidly controlled in SMs, and correlated with an increased expression of PD‐1, the gene encoding the indoleamine 2,3‐dioxygenase (both involved in immunoregulatory mechanisms), and the preservation of CD4 T cell numbers in mucosa and lymphoid tissue homeostasis [9, 10, 11]. Thus, one particularity of SIV infection in SMs is the apparent absence of chronic immune activation, a consequence of which HIV and SIV infection in pathogenic hosts leads to the development of AIDS [3, 12]. The absence of such chronic activation in SMs is thought to be due, in part, to a lack of significant microbial translocation [13], reduced CCR5 expression on CD4+ T cells [14] and reduced activation of innate cells including natural killer (NK) cells and plasmacytoid dendritic cells (pDCs) [15, 16]. Whether activation of monocyte/macrophages and expansion of monocytes that contribute to pathogenesis and are targets of HIV and SIV occurs in SMs has not been studied.

We and others have reported that monocyte expansion during HIV infection in humans and SIV in RMs is predictive of disease progression and histopathogenesis [17, 18, 19, 20]. Blood monocytes are a heterogeneous population of cells. In humans and non‐human primates, three monocyte subsets are identified based on differential expression of CD14 and CD16, including (1) “classical” CD14^++^CD16^−^ monocytes, (2) “intermediate” CD14^++^CD16+ monocytes, and (3) “non‐classical” CD14^+^CD16^++^ monocytes [21, 22, 23]. The presence of the latter two CD16+ monocytes correlates with progression to AIDS pathogenesis. Monocytes play a role in myeloid cell ontogeny as precursors of macrophages and DCs and are also key players in inflammatory responses to microorganisms as innate effectors [24, 25]. Recently, we showed that the recruitment rate of monocytes from bone marrow into blood, measured by BrdU labeling, was increased as early as 8 days post‐infection and correlated with the rapid progression of AIDS and the severity of SIV encephalitis [17, 26]. Further, we found that elevated levels of soluble CD163 (sCD163) shed by activated monocyte/macrophages were better correlates of AIDS than plasma levels of LPS or sCD14 [17, 27]. Indeed, sCD163 and BrdU in SIV‐infected monkeys predict the rate of development to AIDS and correlate with the degree of tissue pathogenesis [17]. In addition, plasma levels of sCD163 positively correlated with the expansion of CD14^++^CD16^+^ monocytes and a decreased surface expression of CD163 on monocytes during HIV and SIV infection prior to and after effective ART [17, 27]. Thus, sCD163 is a sensitive marker of monocyte expansion and activation, and HIV replication that may be informative about innate immune response to SIV and HIV infection and pathogenesis in SMs.

In light of the key role of monocytes in HIV inflammation response and pathogenesis, we sought to determine if a differential response of monocyte/macrophages to SIV infection in SMs could contribute to the low level of immune activation and the absence of AIDS pathogenesis. To test this, we quantified sCD163 in plasma and analyzed the frequencies and the activation of monocytes in naturally SIV‐infected SMs. These results were compared to data from SIV‐infected RMs with AIDS.

## 2 Methods

### 2.1 Animals

Five uninfected and 12 naturally SIV‐infected sooty mangabeys ( *Cercocebus atys* ) housed at the Emory National Primate Research Center (ENPRC) were used in this study. As controls, we also used 23 uninfected RMs, 3 non‐CD8‐depleted, chronically SIV‐infected rhesus macaques, and 5 CD8‐lymphocyte‐depleted, chronically SIV‐infected rhesus macaques ( *Macaca mulatta* ) [28, 29]. At least one 5 mL whole blood sample was collected in ethylenediaminetetraacetic acid (EDTA) for all flow cytometricanalyses. In addition to these animals, flow data from different sets of SMs (*n* = 22 uninfected, *n* = 10 infected) and RMs (*n* = 6 uninfected, *n* = 6 infected) from a previously published study were also used to compare naturally SIV‐infected SMs and experimentally infected RMs (Figure S1) [6].

### 2.2 Flow Cytometry Analysis of Lymphocytes and Monocytes

*Antibodies*: The following cocktails of monoclonal antibodies were used to identify subsets of blood lymphocytes and monocytes: (1) “lymphocyte cocktail” comprising anti‐CD95‐FITC (clone DX2), anti‐CD25‐PE (clone M‐A251), anti‐CD16‐PerCP‐Cy5.5 (clone 3G8), anti‐CCR5‐APC (clone 3A9), anti‐CD3‐Alexa700 (clone SP34‐2), anti‐CD20‐APC‐Cy7 (clone L27), anti‐CD14‐Pacific blue (clone M5E2) (all from BD Bioscience, San Jose, CA), anti‐HLA‐DR‐ECD (clone Immu‐357, Beckman Coulter, Miami, FL), anti‐CD28‐PE‐Cy7 (clone CD28.2, eBioscience, San Diego, CA), anti‐CD4‐Qdot605 (clone S3.5, provided by Dr. K. Reimann, NIH Nonhuman Primate Reagent Resource) and anti‐CD8‐Qdot655 (clone 3B5, Invitrogen, Carlsbad, CA); (2) “monocyte cocktail” comprising anti‐64‐FITC (clone 10.1), anti‐CD86‐PE‐Cy5 (clone 2331), anti‐CD16‐PE‐Cy7 (clone 3G8), anti‐CD11b‐Alexa700 (clone ICRF44), anti‐CD3‐APC‐Cy7 (clone SP34‐2) and CD20‐APC‐Cy7 (clone L27), anti‐CD14‐Pacific blue (clone M5E2) (all from BD Bioscience), anti‐CCR2‐PE (clone 48 607), anti‐CCR8‐APC (clone 19 170) (both from R&D systems, Minneapolis, MN), anti‐HLA‐DR‐ECD (clone Immu‐357, Beckman Coulter), anti‐CD163‐PerCP‐Cy5.5 (clone GHI/61, BioLegend, San Diego, CA), and anti‐CD4‐Qdot605 (clone S3.5, NIH Nonhuman Primate Reagent Resource). *Staining*: Erythrocytes in 100 μL of whole blood were lysed using Immunoprep reagent on a T‐Q prep machine (Beckman‐Coulter, Fullerton, CA). The remaining leukocytes were washed with phosphate‐buffered saline (PBS) containing 2% fetal bovine serum (FBS) and incubated with a pre‐mixed antibody cocktail described above for 15 min at room temperature in the dark. Stained cells were washed with PBS‐2% FBS and resuspended in freshly prepared 1% paraformaldehyde (PFA). For intracellular staining of MAC387 and CD68, leukocytes were fixed and permeabilized using a Fix/Perm kit according to the manufacturer's protocol (BD Bioscience), washed, and stained for 30 min in the dark with anti‐MAC387‐FITC (clone MAC387, AbD serotec, Raleigh, NC) or anti‐CD68‐FITC (clone KP‐1, Dako, Carpinteria, CA). Fixed cells were analyzed on a BD FACS Aria flow cytometer (BD Biosciences) as previously described [30]. A minimum of one million total events were collected for analysis. Data were analyzed using FlowJo software (version 7; Treestar, Ashland, OR).

### 2.3 sCD163 ELISA

Soluble CD163 (sCD163) plasma levels were quantified by ELISA according to the manufacturer's protocol (Trillium Diagnostics, Brewer, ME).

### 2.4 Plasma SIV RNA

Plasma SIV RNA was quantified using real‐time PCR as previously described [31]. SIV virions were pelleted from 0.5 mL of plasma by centrifugation at 20 000 g for 1 h. The fluorescently labeled, real‐time PCR probe employed contained a nonfluorescent quencher, BHQ‐1 (Biosearch Technologies Inc.), at its 3′ end instead of the fluorescent quencher TAMRA (6‐carboxy‐tetramethyl‐rhodamine). The threshold sensitivity was 100 copy Eq/mL, with an average interassay coefficient of variation of less than 25%.

### 2.5 Variable Importance Analysis

Phenotyping data from SMs were analyzed to evaluate monocyte subset distribution and activation status in uninfected (*n* = 6) and SIV‐infected SMs (*n* = 12), and in SIV‐infected RM (*n* = 3) and SIV‐infected SMs (*n* = 12). Flow cytometry‐based frequencies and phenotypic marker expression of CD14^+^ monocytes and CD14^+^CD16^+^ subsets were quantified, including activation and trafficking markers (CD11b, CCR2, CCR5, CCR8, CD163, HLA‐DR, CD86, Mac387, CD68, and CD44v6) as previously described [20]. Plasma sCD163 and viral load were included as systemic immune activation and infection markers. Raw (non‐normalized, non‐log‐transformed) data were analyzed in R (version 4.4.2). Heatmap visualization was performed using the pheatmap package to assess global patterns of monocyte phenotypes across groups. Supervised multivariate analyses, including Partial Least Squares–Discriminant Analysis (PLS‐DA) and sparse PLS‐DA (sPLS‐DA), were conducted using the mixOmics package to identify features discriminating uninfected and SIV‐infected SMs. Random Forest classification analysis was performed using the **r**andomForest package (ntree = 5 000) to rank variable importance and identify key predictors of group separation. Model performance and variable importance measures were used to determine the most discriminative monocyte‐associated markers.

### 2.6 Statistical Analysis

Nonparametric Mann–Whitney t‐test or Student's t‐test was used for comparisons of cell percentages between uninfected and SIV‐infected animals. Differences were considered statistically significant with *p* values < 0.05. Correlations were determined using Spearman's non‐parametric test with two‐tailed *p* values < 0.05 considered significant. Statistical analyses were computed with Prism software (version 5.02; GraphPad Software, La Jolla, CA).

## 3 Results

### 3.1 SIV‐Infected Sooty Mangabeys Are Characterized by Depletion of CD4+ Effector T Lymphocytes and Lack of CCR5 Expression on Lymphocytes

**FIGURE 1:**
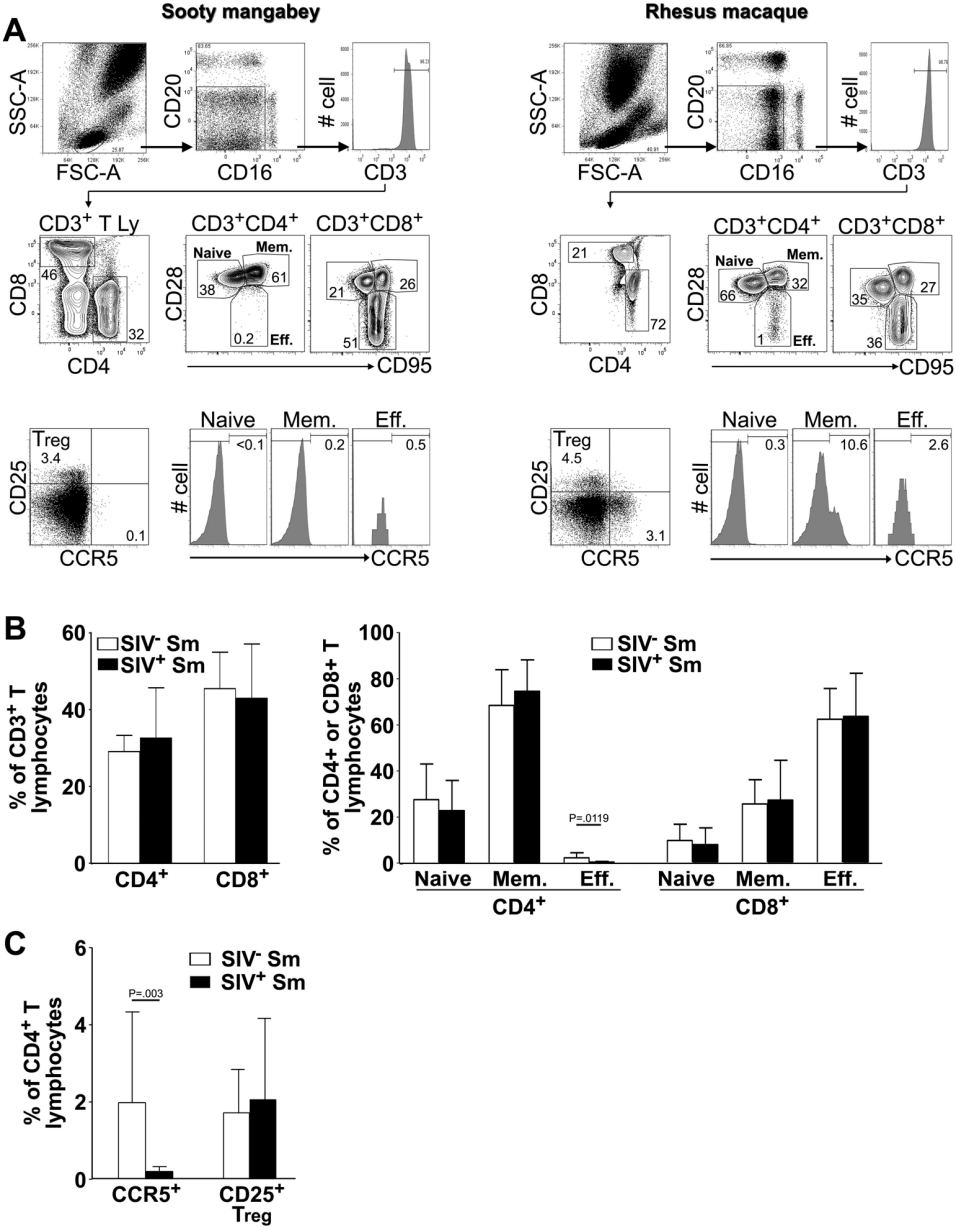
Lack of CD4+ effector T cells and lymphocytic CCR5 expression in naturally infected sooty mangabeys. (A) Comparison of representative lymphocyte gating strategies from one SIV‐infected SM (left) and one SIV‐infected RM (right) is shown. (A) Top row: Lymphocytes were first gated according to their forward (FSC) and side (SSC) scatter properties. CD20+ B lymphocytes and CD16+ NK cells were excluded from the analysis to obtain a homogeneous population of CD3+ T cells (histogram). (A) Middle row: CD4+ and CD8+ T lymphocytes were gated from CD3+ T lymphocytes. From each population, subsets of naïve CD95‐CD28+, memory CD95 + CD28+, and effector CD95 + CD28‐ lymphocytes were identified. (A) Bottom row: Representative dot plot showing the gating of regulatory CD4+ T lymphocytes identified by co‐expression of CD25. (B) Histograms represent percentage of CD3+CD4+ and CD3+CD8+ lymphocytes (left) and CD4+ and CD8+ naive, memory and effector T lymphcoytes. (C) The median fluorescence intensity (MFI) of surface expression of CCR5 and CD25 on CD4+ lymphocyte subsets. Data for B and C are expressed as percentages of positive cells.

We first analyzed lymphocyte populations in naturally infected SMs and compared them to lymphocyte populations in experimentally, chronically infected RMs to confirm the differences previously reported between SMs and pathogenic hosts like RMs (Figure 1). We did not find any significant differences in the percentages of total CD4+ and CD8+ T lymphocytes in the infected SMs compared to uninfected SMs or infected RMs (Figure 1A,B). However, we observed CD3 + CD4‐CD8‐ T cells in SM and not in SIV‐infected RMs (Figure 1A). Overall, SIV‐infected SMs had similar percentages of naïve and memory CD8+ T lymphocytes but significantly higher percentages of effector CD8+ T lymphocytes (mean ± SD; 64% ± 19%) compared to SIV‐infected RMs (42% ± 5%) (*p* = 0.04, Mann–Whitney test) (Figure 1A,B Table 1). We found significant differences in the frequencies of CD4+ T lymphocyte subsets between infected SMs and RMs (Figure 1, Table 1), where the percentages of CD4+ naïve T cells were significantly lower in infected SMs (23% ± 13%) than in infected RMs (77% ± 12%) (*p* = 0.01). The percentages of CD4+ memory T cells were significantly higher in infected SMs (75% ± 14%) than in infected RMs (19% ± 12%) (*p* = 0.01). CD4+ effector T cells were significantly depleted in infected SMs (uninfected SMs, 2.6% ± 2.3% vs. infected SMs, 0.4% ± 0.3%; *p* = 0.01) (Figure 1A, Table 1) but were not in the SIV‐infected RMs (2.2% ± 2%). Moreover, CD4 + CCR5+ T cells were significantly lower in infected SMs than in uninfected SMs (*p* = 0.002) (Figure 1C, Table 1). To further determine whether SIV infection alters chemokine receptor expression and lymphocyte trafficking profiles in sooty mangabeys, we evaluated the expression of CCR5, CXCR4, and CD62L by flow cytometry (Figure S1A). We found that CCR5 and CXCR4 expression were not significantly different between uninfected and SIV‐infected animals (*p* = 0.1308 and *p* = 0.7783, respectively; unpaired t‐test). Similarly, CD62L expression did not differ significantly between uninfected and SIV‐infected SMs, indicating preserved lymphocyte homing capacity in this natural host. In contrast, CD62L expression was significantly reduced in SIV‐infected rhesus macaques compared to uninfected (*p* = 0.0022) (Figure S1D), highlighting a divergence in the regulation of lymphocyte trafficking between pathogenic and non‐pathogenic hosts. Additionally, CD62L expression showed an association with age across animals, suggesting that variability in lymphocyte trafficking markers may be influenced by host factors rather than SIV infection. Together, these findings indicate that key chemokine receptors and trafficking molecules remain stable during SIV infection in sooty mangabeys, whereas pathogenic infection in rhesus macaques is associated with disruption of lymphocyte homing phenotypes. We did not find significant differences between the percentages of CD4 + CD25+ T regulatory cells in uninfected and infected SMs (Table 1). Altogether, these results are in agreement with previously published data demonstrating the presence of CD3 + CD4‐CD8‐ T lymphocytes and a lack of effector CD4+ T cells and CCR5 expression as features distinguishing SIV infection in SMs from pathogenic RMs.

**TABLE 1:** Percentages of lymphocytes in uninfected and SIV‐infected sooty mangabeys vs. rhesus macaques.

	Sooty mangabeys (SMs)			Rhesus macaques (RMs)		SIV + SMs vs. SIV + RMs
	SIV‐	SIV+	SIV‐ vs. SIV +	SIV‐	SIV + ^b^	
	*n* = 5	*n* = 12	*p* value^a^	*n* = 3	*n* = 3	*p* value^a^
CD3 + CD4+ T lymphocytes	30.1 ± 3.9	32.6 ± 13.1	ns	52.7 ± 5.4	43.9 ± 24.6	ns
Naïve CD4 + CD95‐CD28+	30.9 ± 14.6	22.7 ± 13.2	ns	58.9 ± 17.0	76.9 ± 11.6	0.01
Effector CD4 + CD95 + CD28−	2.6 ± 2.3	0.4 ± 0.3	0.007	5.3 ± 1.4	2.2 ± 2.0	0.01
Memory CD4 + CD95 + CD28+	64.5 ± 13.5	74.6 ± 13.7	ns	34.0 ± 16.5	18.6 ± 12.1	0.01
CD4 + CCR5+ T lymphocytes	2.3 ± 2.4	0.2 ± 0.1	0.002	5.2 ± 2.9	1.3 ± 1.7	ns
Regulatory CD4 + CD25+	1.8 ± 1.2	2.1 ± 2.1	ns	3.4 ± 2.4	3.3 ± 1.0	ns
CD3 + CD8+ T lymphocytes	42.8 ± 7.8	43.0 ± 14.1	ns	35.6 ± 3.2	48.2 ± 23.7	ns
Naïve CD8 + CD95‐CD28+	10.6 ± 7.4	7.8 ± 7.3	ns	51.5 ± 14.8	21.5 ± 18.0	ns
Effector CD8 + CD95 + CD28−	59.5 ± 13.1	63.6 ± 18.8	ns	31.0 ± 9.2	41.9 ± 5.2	ns
Memory CD8 + CD95 + CD28+	27.0 ± 11.1	27.2 ± 17.3	ns	15.6 ± 8.4	21.0 ± 6.9	ns

*Note:* Data are mean ± SD of cell percentages.

Abbreviations: RMs, rhesus macaques; SMs, sooty mangabeys.

^a^ Non‐parametric Mann–Whitney test comparing lymphocyte percentages between SIV‐ and SIV+ animals and between infected SMs and RMs. *p* values < 0.05 were considered significant.

^b^ No significant differences in lymphocyte percentages were found between SIV‐ vs. SIV+ rhesus macaques.

### 3.2 Lack of Elevated sCD163 in Plasma of SIV‐Infected Sooty Mangabeys

**FIGURE 2:**
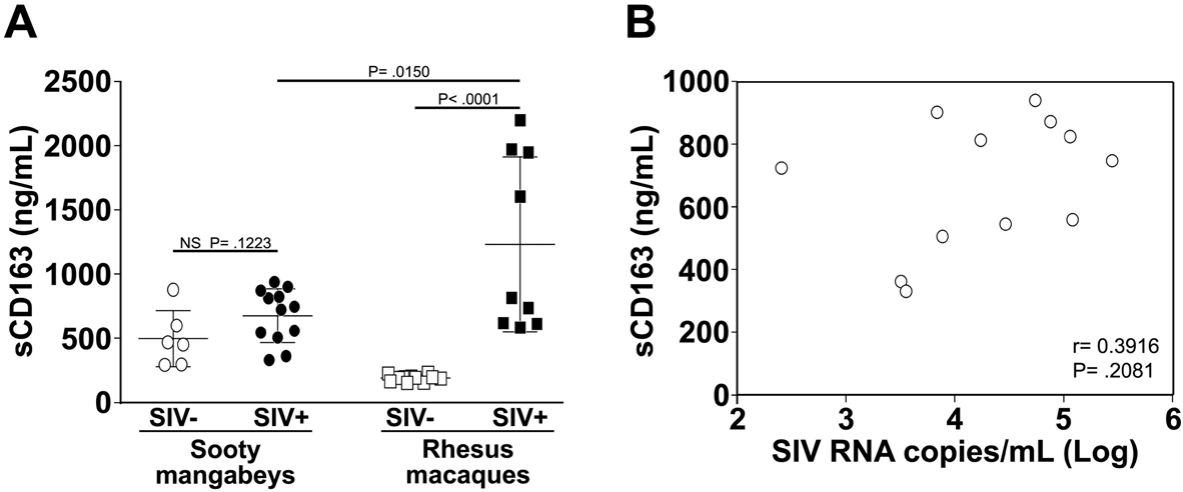
Soluble CD163 levels in plasma are not increased in SIV‐infected sooty mangabeys. (A) sCD163 levels were quantified in plasma from uninfected and SIV‐infected SM, and uninfected and CD8 T cell‐depleted SIV‐infected RM with AIDS. To test for significant differences between the plasma levels of sCD163 in uninfected and SIV‐infected SM or RM, a Mann–Whitney non‐parametric test or unpaired t‐test was used depending on whether or not data samples passed the normality test. Values of *p* < 0.05 were considered statistically significant. (B) Plasma levels of sCD163 measured in SIV‐infected SM did not correlate with plasma virus. Spearman correlation was used. The coefficient of correlation and the *p* value (two‐tailed) are shown.

We previously reported that elevated plasma sCD163, made exclusively by myeloid cells including monocyte/macrophages, was significantly increased in SIV‐infected RMs and correlated with plasma virus, increased blood monocyte turnover, and severity of SIV pathogenesis [17]. We also showed that sCD163 is a marker of HIV activity in early and chronic infection that decreases with successful ART [27]. Basal plasma sCD163 in uninfected SMs was higher compared to uninfected RM (*p* = 0.0009, Mann–Whitney test) but both were in the range of previous data from uninfected humans and monkeys [27]. Soluble CD163 in SIV‐infected SM (676 ng/mL±209 ng/mL) was not significantly different from uninfected SM (498 ng/mL±219 ng/mL) (*p* = 0.12, Mann Whitney test) (Figure 2A). This contrasted with the significant increase of sCD163 in RMs with SIV infection (uninfected RMs, 191 ng/mL±24 ng/mL vs. infected RMs, 1231 ng/mL±686 ng/mL; *p* < 0.0001, unpaired t‐test). In contrast to HIV infection, where we found a significant correlation between plasma virus and elevated sCD163 [27], plasma sCD163 in SIV‐infected SMs did not correlate with plasma virus (Spearman's *r* = 0.39, *p* = 0.2) (Figure 2B). These results highlight a significant difference in the innate response in non‐pathogenic vs. pathogenic hosts and suggest a lack of monocyte/macrophage activation in naturally SIV‐infected SMs.

### 3.3 Sooty Mangabeys Exhibit Marked Differences in Frequencies of Monocyte Subsets Compared to Rhesus Macaques

**FIGURE 3:**
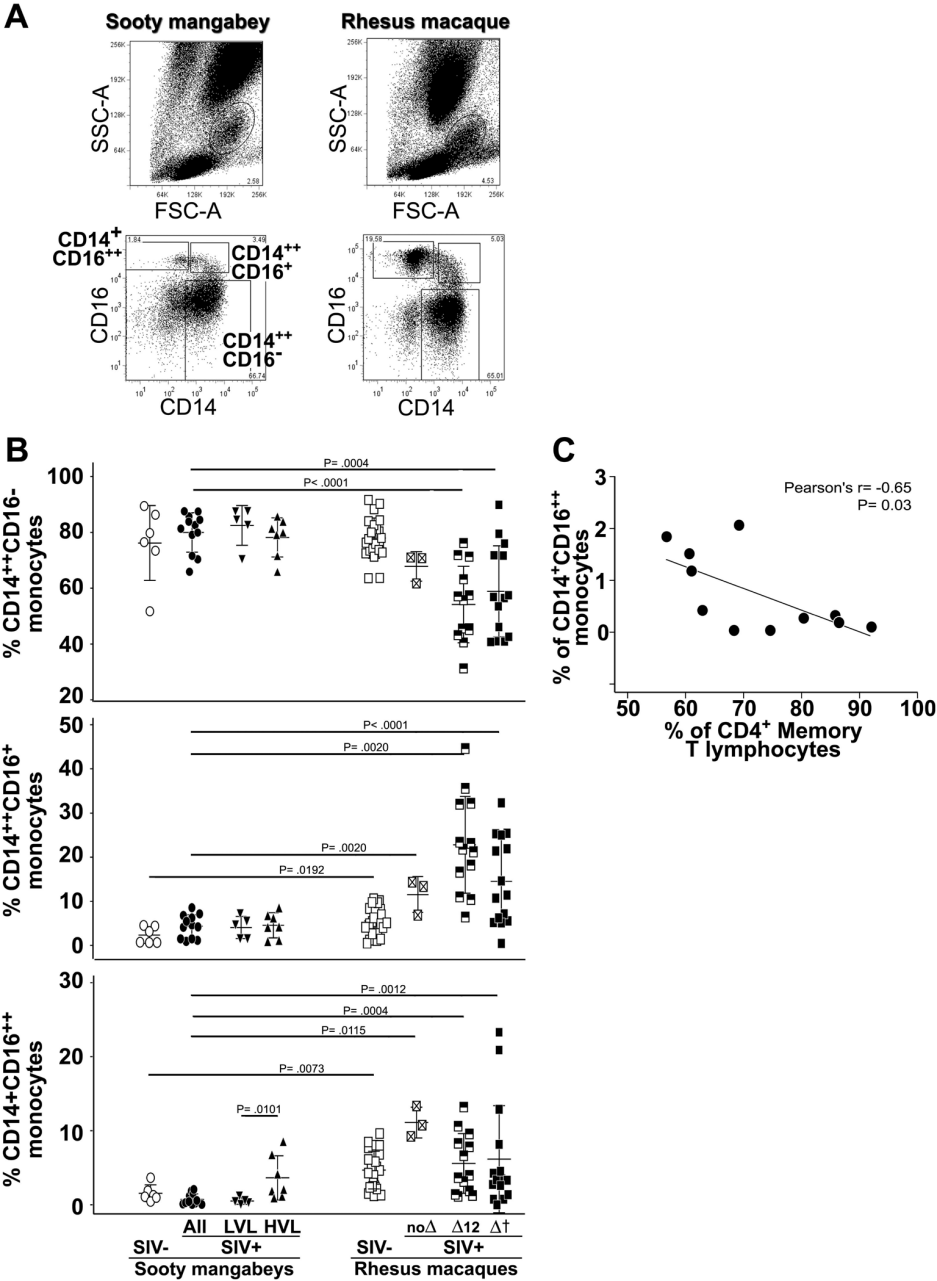
Frequencies of monocyte subsets in SIV‐infected sooty mangabeys and rhesus macaques. (A) Comparison of representative monocyte gating strategies from one SIV‐infected SM (left) and one SIV‐infected RM (right) is shown. Monocytes were first gated according to their FSC/SSC properties. After exclusion of CD3+ T lymphocytes, CD20+ B cells, and CD16+ NK cells (not shown), three monocyte subsets were distinguished according to their expression of CD14 and CD16: CD14++CD16‐ “classical” monocytes and two subsets of CD16+ monocytes defined as CD14++CD16+ “intermediate” and CD14 + CD16++ “non‐classical” monocytes. (B) Scatter plots representing percentages of CD14++CD16‐ (upper graph), CD14++CD16+ (middle graph), and CD14 + CD16++ monocytes (lower graph) from SM and RM are shown. Percentages of monocyte subsets were determined in uninfected (SIV‐) and SIV‐infected (SIV+) SM. Frequencies of monocyte subsets were also determined in uninfected (SIV‐), non‐CD8‐depleted SIV‐infected (SIV+) RM and CD8+ T lymphocyte‐depleted SIV‐infected RM at peak viremia (i.e., 12 days post‐infection, Δ 12) and terminally with AIDS (Δ†) at euthanasia between 60 and 70 dpi. To test for significant differences between monocyte percentages among the different groups of animals, a non‐parametric t‐test was used. Values of *p* < 0.05 that were considered statistically significant are shown. Horizontal bars indicate means ± SD. (C) Percentages of CD14 + CD16++ monocytes negatively correlated with percentages of CD4+ memory T lymphocytes in SIV‐infected SM. Pearson correlation was used. Percentages of CD14++CD16‐ monocytes positively correlated with percentages of CD8+ memory T lymphocytes in SIV‐infected SM. Spearman correlation was used. The coefficient of correlation and the *p* value (two‐tailed) are shown.

Soluble CD163 is shed exclusively from activated monocyte/macrophages, as CD14++CD16+ monocytes have the highest expression of CD163 and presumably the highest shedding of CD163 [17, 27, 32]. Thus, we further investigated potential differences in the monocytic compartment between non‐pathogenic and pathogenic hosts by the analysis of blood monocyte subsets. We determined the relative percentages of CD14++CD16‐, CD14++CD16+, and CD14 + CD16++ monocytes in uninfected and SIV‐infected SMs. SMs were compared to SIV‐infected RMs and CD8+ T lymphocyte‐depleted SIV‐infected RMs at peak viremia (12 days post‐infection, dpi) and terminally with AIDS at euthanasia between 60 and 70 dpi (Figure 3). We found no significant differences in the frequencies of CD14++CD16‐, CD14++CD16+, and CD14 + CD16++ monocytes between uninfected and SIV‐infected SMs (Figure 3A,B). The percentages of CD14++CD16‐ monocytes in infected SMs were higher than in infected RMs (*p* = 0.05, Mann–Whitney test) and more so in CD8‐depleted SIV‐infected RMs at 12 dpi and with terminal AIDS (*p* < 0.0001 and *p* = 0.0004, respectively; unpaired t‐test) (Figure 3B). Conversely, the percentages of activated CD14^++^CD16^+^ and CD14^+^CD16^++^ monocytes in SIV‐infected SMs (4.3% ± 2.6% and 0.72% ± 0.74%, respectively) were significantly lower than in infected RMs (12% ± 4% and 11% ± 2%, respectively) and in CD8‐depleted SIV‐infected RMs at 12 dpi (23% ± 11% and 5.6% ± 4.0%, respectively) and terminally with AIDS (15% ± 10% and 6.2% ± 7.2%, respectively) (Figure 3B). Finally, the percentages of CD16+ monocytes were significantly lower in SMs than in RMs without SIV infection (*p* < 0.02) (Figure 2B). These results show that, in contrast with pathogenic hosts, there is no expansion of activated CD16+ monocytes in SMs.

Interestingly, in contrast with all RMs and uninfected SMs, CD4+ memory T cells were negatively correlated with CD14^+^CD16^++^ monocytes (Pearson's *r* = −0.65, *p* = 0.03) (Figure 3C) while CD8+ memory T cells were positively correlated with CD14++CD16‐ monocytes (Spearman's *r* = 0.60, *p* = 0.04), suggesting a role of memory T cells in the control of the frequencies of activated monocytes in SIV‐infected SMs.

### 3.4 Low Levels of Monocyte Activation in SIV‐Infected Sooty Mangabeys

**FIGURE 4:**
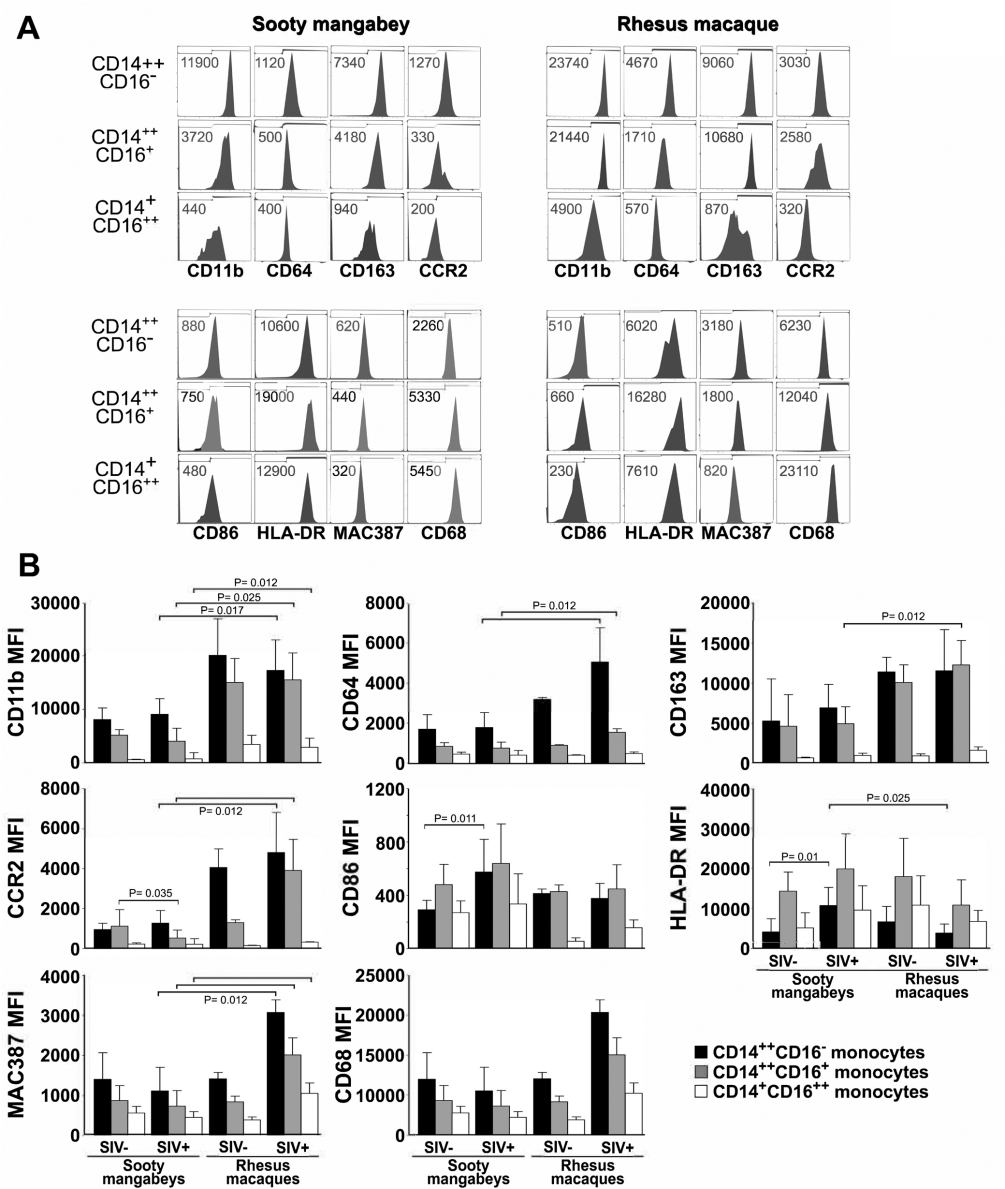
Expression pattern of monocytic markers in sooty mangabeys. Bar charts are mean ± SD of monocytic markers expressed on CD14++CD16‐ (black histograms), CD14++CD16+ (gray histograms) and CD14 + CD16++ (white histograms) monocytes from uninfected (SIV‐, *n* = 5) and SIV‐infected (SIV+, *n* = 12) SM, and uninfected (SIV‐, *n* = 3) and non CD8‐depleted SIV‐infected RM (SIV+, *n* = 3) are shown. To test for significant differences between expression levels of markers in the different groups of animals, a Mann–Whitney non‐parametric test was used. Values of *p* < 0.05 that were considered statistically significant are shown.

We analyzed the expression of markers that are relevant for the monocyte inflammation process, including CD163, CD11b, CD64, CD86, HLA‐DR, MAC387, and CCR2, co‐receptors for HIV and SIV, including CD4 and CCR5, and maturation marker CD68 (Figure 4). Expression of CD163, CD11b, CD64, MAC387, and CD4 on monocytes from SIV‐infected SMs was significantly lower compared to SIV‐infected RMs (Figure 4), suggesting that monocytes from SIV‐infected SMs were, overall, less activated. Expression of CCR5 and CD68 on monocytes was comparable between infected or uninfected SMs and RMs (not shown). Compared to uninfected SMs, expression of HLA‐DR and CD86 on CD14++CD16‐ monocytes was significantly increased in infected SMs (*p* < 0.02), while CD4 expression on CD14 + CD16++ monocytes was significantly decreased in SIV‐infected SMs (*p* = 0.02). We found no differences between uninfected and SIV‐infected SMs with regard to expression of CD163, CD11b, CD64, CD68, and MAC387 on the three subsets of monocytes (Figure 4). CD64 expression on CD14++CD16‐ monocytes from SIV‐infected SMs was positively correlated with plasma virus (Spearman's *r* = 0.60, *p* = 00.039) while the expression of CD163 and CD86 on CD14 + CD16++ monocytes were negatively correlated with plasma virus (Spearman's *r* = −0.63, *p* = 0.03 and *r* = −0.65, *p* = 0.03, respectively). In contrast to what we found with HIV‐infected humans and SIV‐infected RMs, we did not find any correlation between plasma sCD163 detected in SIV‐infected SMs and the surface expression of CD163 on activated CD14++CD16+ monocytes. These results suggest that the two CD16+ monocyte subsets, whose percentages in SMs without AIDS are lower than in RMs that developed AIDS, do not display markers associated with activation, in contrast with monocytes in RMs.

### 3.5 Distinct Monocyte Phenotypic Signatures Characterize Natural and Pathogenic SIV Infection

To investigate how SIV infection influences monocyte/macrophage activation in natural vs. pathogenic hosts, we performed multivariate and machine learning‐based analyses on circulating monocyte phenotypes from uninfected and SIV‐infected SMs, as well as SIV‐infected RMs.

**FIGURE 5:**
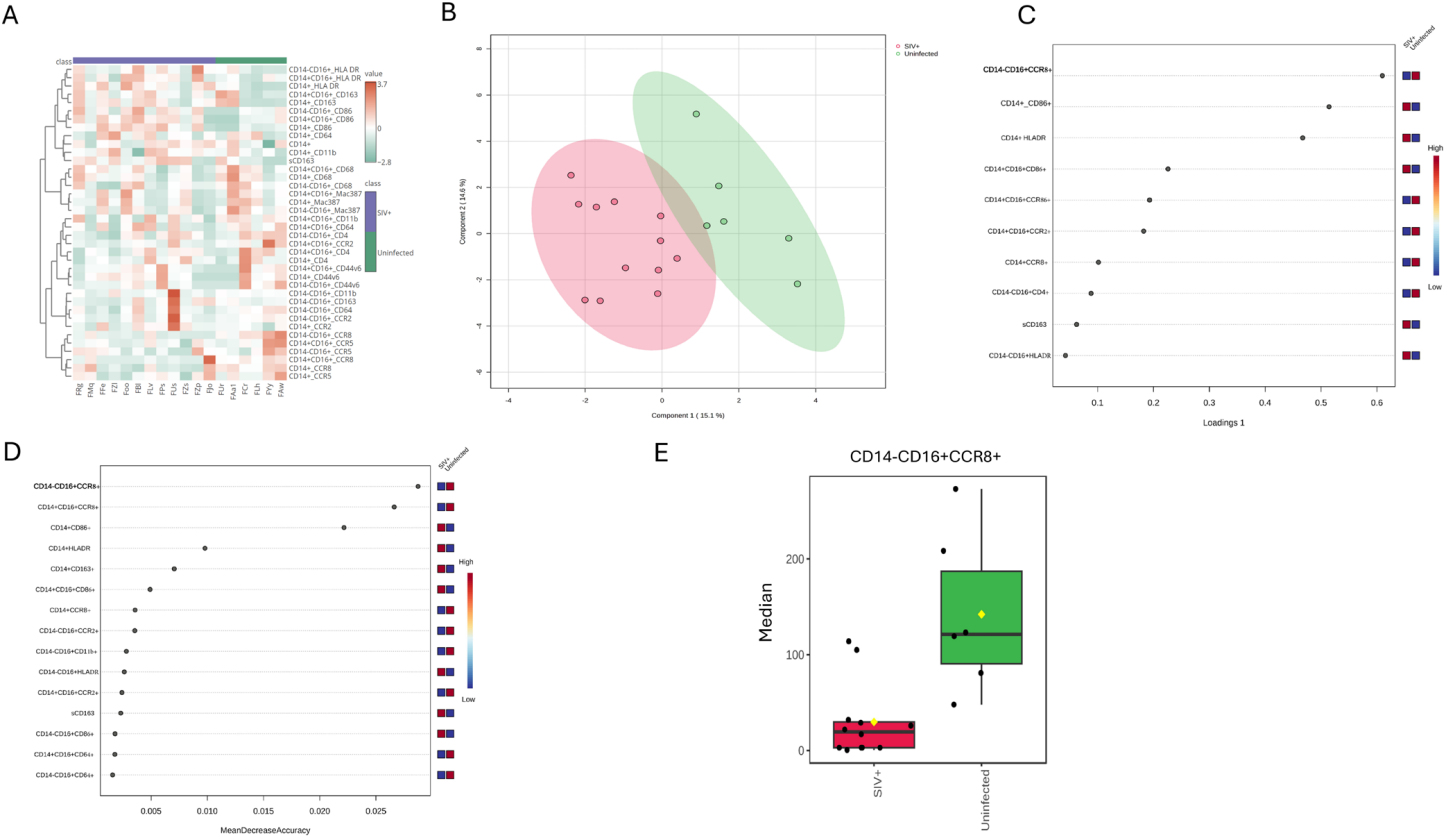
Monocyte phenotypic signatures distinguish uninfected and SIV‐infected sooty mangabeys. (A) Heatmap analysis of circulating monocyte phenotypes and activation markers in uninfected (*n* = 6) and SIV‐infected sooty mangabeys (*n* = 12). Raw flow cytometry–derived parameters, including frequencies of CD14^+^ and CD14^+^CD16^+^ monocytes and expression of activation and trafficking markers (CD11b, CCR2, CCR5, CCR8, CD163, HLA‐DR, CD86, Mac387, CD68, CD44v6, and CD4), together with plasma sCD163 and viral load, were analyzed in R using hierarchical clustering and visualized as a heatmap. (B) Partial Least Squares–Discriminant Analysis (PLS‐DA) and sparse PLS‐DA (sPLS‐DA) were performed using the *mixOmics* R package to evaluate group separation based on monocyte phenotypic profiles. (C–E) Variable importance analysis using Random Forest classification (*randomForest* package, ntree = 5000) identifying key discriminative monocyte‐associated markers distinguishing uninfected and SIV‐infected sooty mangabeys.

Heatmap clustering revealed partial segregation between uninfected and SIV‐infected SMs, although substantial inter‐individual variability was observed (Figure 5A). In contrast, comparison between SIV‐infected SMs and RMs demonstrated a more pronounced separation (Figure S2A). Supervised PLS‐DA and sparse PLS‐DA analyses further supported these findings, showing partial separation between uninfected and SIV‐infected SMs and clearer discrimination between SIV‐infected SMs and RMs (Figure 5B and Figure S2B).

To identify key drivers of these differences, Supervised PLS‐DA, sparse PLS‐DA, and Random Forest analyses were performed. Among all evaluated parameters, CD14^+^CD16^+^CCR8^+^ monocytes emerged as the most important variable distinguishing uninfected from SIV‐infected SMs, suggesting a role for CCR8‐associated trafficking or tissue‐homing in the regulated immune response of natural hosts (Figure 5C–E). In contrast, CD14^+^CD16^+^CD11b^+^ monocytes were identified as the strongest discriminator between SIV‐infected SMs and SIV‐infected RMs, indicating that CD11b‐associated activation and adhesion properties are key features of pathogenic SIV infection (Figure S2C–E).

## 4 Discussion

Here, we report the absence of expansion and activation of monocytes in naturally SIV‐infected SMs compared to pathogenic hosts, RMs. The first marked difference that we observed between SMs and RMs is the lack of significant increase in plasma levels of sCD163 in SMs that do not develop AIDS despite high viremia. By comparison, SIV‐infected RMs that progressed to AIDS showed a 6‐fold increase in their plasma levels of sCD163 compared to uninfected controls. CD163 is a scavenger receptor selectively expressed on monocytes/macrophages [33]. It has a role in the clearance of hemoglobin and in the regulation of inflammatory responses by stimulating the secretion of anti‐inflammatory cytokines [34]. CD163 is shed in a soluble form following monocyte/macrophage activation [35, 36]. sCD163 serves as a biomarker of HIV activity and AIDS pathogenesis [27]. sCD163 has also been shown to be increased in various inflammatory clinical diseases such as atherosclerosis, rheumatoid arthritis, multiple sclerosis, or psoriasis, but it does not correlate absolutely with these diseases, as other cells in addition to macrophages are involved in pathogenesis [37, 38]. We have recently reported that plasma levels of sCD163 in pathogenic hosts were significantly elevated following SIV or HIV infection and correlated with percentages of activated CD14 + CD16+ monocytes and activated CD8 + HLA‐DR + CD38+ T lymphocytes, underscoring sCD163 as a marker of HIV activity in early and chronic infection [17, 27]. We also showed that plasma levels of sCD163 correlate with the turnover of BrdU+ monocytes recruited from bone marrow during SIV infection and with the severity of SIV‐associated encephalitis [17]. In humans, sCD163 is positively correlated with plasma virus and is negatively correlated with surface expression levels of CD163 on monocytes [27]. Here we show that plasma levels of sCD163 from SIV‐infected SM do not correlate with plasma virus, percentages of monocyte or lymphocyte subsets, or with the expression level of surface‐bound CD163 on monocytes. The absence of high levels of plasma sCD163 in SIV‐infected SM despite high viremia might be explained by the lack of chronic inflammation reported in SM, which distinguishes them from SIV‐infected RM or HIV‐infected humans [39, 40]. It is interesting to note these differences, which underscore the role of innate immunity, specifically monocyte/macrophages and activation levels with respect to SIV infection that are immunologically linked to the acquired immune system, with reflective differences in the lack of lymphocyte activation in SM compared to RM. This also seems to correlate directly with the lack of pathogenesis in SM vs. RM that we report here. Several studies investigated the role of innate immunity in SIV infection of non‐pathogenic hosts and suggested that pDCs and NK cells were involved in the innate immune response [15, 16]. It has been reported that the levels of type I IFN secreted by pDCs as part of the anti‐viral response in non‐pathogenic hosts, although increasing during acute infection, were decreased with chronic infection. Acute infection in SMs and RMs is associated with a significant upregulation of IFN‐stimulated genes, but only SMs are able to rapidly resolve it [10]. Another particularity of non‐pathogenic SIV infection is the absence of microbial translocation [13, 41]. The leakage of microbial components from the gut to the periphery that occurs during HIV and SIV infection in pathogenic hosts is thought to contribute to activation of immune responses via the crosslinking of TLR4 with LPS [13, 42]. In SMs, the intestinal barrier remains intact despite SIV infection and therefore explains in part the low plasma levels of LPS. This may explain the lack of monocyte/macrophage activation and CD163 shedding since LPS stimulates sCD163 shedding in vitro [36]. Interestingly, we did not find a correlation in RMs and humans between LPS in plasma and sCD163, suggesting that additional stimuli are involved in sCD163 shedding in vivo.

We found that uninfected and SIV‐infected SMs were characterized by significantly low percentages of CD16+ monocytes, both the “intermediate” CD14++CD16+ and the “non‐classical” CD14 + CD16++ monocyte subsets, which is in contrast with monocyte expansion detected during HIV and SIV infection that can predict the progression to AIDS and AIDS dementia [17, 18, 26, 43]. Indeed, our studies with BrdU and sCD163 in SIV‐infected monkeys and HIV infected humans show that both markers predict the rate of AIDS development and are linked to the degree of non‐calcified vulnerable cardiac plaque and the degree of HAND severity in HIV infected individuals on ART. Based on in vivo studies in rodents, it has been suggested that classical CD14++CD16‐ human monocytes resemble inflammatory Gr1+ murine monocytes specialized in TNF‐α production [44]. On the other hand, human CD16+ monocytes are closer to Gr1‐ monocytes, suggesting that “non‐classical” CD14 + CD16++ monocytes are considered “resident” monocytes that constitutively home to tissues and are mobilized with inflammatory conditions [44, 45]. CD14 + CD16++ monocytes can produce high levels of inflammatory cytokines in the presence of virus [45, 46, 47]. Thus, low frequencies of CD14 + CD16++ monocytes observed in uninfected and infected SM (< 2%) might account for the lack of chronic inflammatory responses and AIDS observed in non‐pathogenic hosts despite high plasma virus. Moreover, CD14++CD16+ monocytes might be the main source of sCD163, as these cells express the highest levels of surface CD163 compared to CD14++CD16‐ and CD14 + CD16++ monocytes [32]. Therefore, one could expect reduced shedding of CD163 in SMs due to the low frequency of CD14++CD16+ monocytes compared to RM. We also found that only the classical CD14++CD16‐ monocytes in SMs have increased expression of activation markers, as illustrated by the upregulation of CD86 and HLA‐DR expression, whereas the CD14++CD16+ and CD14 + CD16++ monocytes were not. As sCD163 is released with activation of monocyte/macrophages, a reduced activation of CD16+ monocytes might also explain the low sCD163 in plasma of SIV‐infected SMs.

In addition to differences in monocyte activation, our analysis of markers on lymphocytes associated with immune cell trafficking revealed a clear divergence between SMs and RMs. While CCR5 and CXCR4 expressions remained stable in SM following SIV infection, consistent with preserved immune homeostasis [48], SIV‐infected RMs exhibited a significant reduction in CD62L expression, reflecting altered lymphocyte trafficking. CD62L (L‐selectin) is a key regulator of lymphocyte homing to secondary lymphoid tissues, and its downregulation is commonly associated with T cell activation and differentiation toward effector phenotypes [49]. The maintenance of CD62L expression in sooty mangabeys suggests that lymphocyte trafficking and tissue homing remain tightly regulated despite high levels of viral replication, whereas its reduction in rhesus macaques is indicative of immune activation and redistribution of lymphocyte subsets characteristic of pathogenic infection. Our data reported here underscore the role of myeloid cells of the innate immune response in differentially activating lymphocytes in SM vs. RM. Furthermore, the observed association between CD62L expression and age suggests that host intrinsic factors, rather than infection alone, contribute to variability in trafficking phenotypes. Together, these findings support the concept that natural hosts maintain immune equilibrium by preserving both receptor expression and trafficking dynamics, thereby limiting immune activation and disease progression. The lack of CCR5 on CD4+ T cells in non‐pathogenic hosts and the presence of double‐negative T cells, lacking CD4 expression, have been attributed to the resistance of SMs to SIV infection [50]. Similarly, the low percentage of CD16+ monocytes and the lower expression of CD4 on monocytes that we found in SMs, infected or not, compared to RMs, could also explain this resistance to SIV infection in tissue macrophages in non‐pathogenic hosts. CD16+ monocytes are thought to be preferentially infected by SIV and are key players in the development of AIDS‐associated encephalitis, as they represent productive viral reservoirs able to transport the virus in the bloodstream to the CNS [51]. Whether subsets of CD16+ monocytes in naturally infected SM from the present study harbored SIV‐RNA and DNA needs to be determined.

We show that this controlled immune state extends to the monocyte compartment, where SIV infection in SMs induces subtle, regulated phenotypic changes rather than broad activation. Multivariate and machine learning analyses identified distinct monocyte signatures associated with infection and disease outcome. Within SMs, SIV infection was primarily associated with CD14^+^CD16^+^CCR8^+^ monocytes, suggesting modulation of trafficking or tissue‐homing functions rather than inflammatory activation [52, 53]. In contrast, pathogenic infection in RMs was characterized by CD14^+^CD16^+^CD11b^+^ monocytes, consistent with enhanced activation, adhesion, and inflammatory recruitment [54, 55]. These findings indicate that the quality of monocyte responses, rather than their magnitude, differentiates non‐pathogenic from pathogenic SIV infection. Overall, our results support a model in which natural hosts maintain functionally regulated innate immune responses, potentially limiting chronic inflammation and disease progression. In contrast, pathogenic hosts exhibit enhanced activation of inflammatory monocyte subsets, which may contribute to immune dysregulation and tissue damage. These findings highlight monocyte functional programming as a key determinant of SIV pathogenesis and a potential target for therapeutic strategies in HIV infection.

We conclude that in contrast with pathogenic hosts such as SIV‐infected RM and HIV‐infected humans, naturally SIV‐infected SMs are characterized by the absence of monocyte expansion and do not increase plasma levels of sCD163 despite high viremia. These are significant differences with pathogenic hosts that could help maintain an inflammation‐free environment in combination with lymphocytes, NK cells, and pDCs and contribute to the lack of chronic immune response and absence of progression to AIDS.

## Funding

This work was supported by the TNBRC P51 Base Grant (P51OD011104) and ENPRC P51 Base Grant (P51OD011132) from the National Institutes of Health and NIH R01NS040237 (KCW) and R01NS126091 (KCW & W‐KK).

## Ethics Statement

The authors confirm that the ethical policies of the journal, as noted on the journal's author guidelines page, have been adhered to and the appropriate ethical review committee approval has been received. The US National Research Council's guidelines for the Care and Use of Laboratory Animals were followed.

## Conflicts of Interest

The authors declare no conflicts of interest.

## Supporting information


**Figure S1:** Characteristics of CCR5, CXCR4, and CD62L in SIV‐infected sooty mangabeys and rhesus macaques. (A) Comparison of CCR5 (A), CXCR4 (B), and CD62L (C) from sooty mangabeys (SMs; *n* = 22 uninfected, *n* = 10 SIV‐infected) and rhesus macaques (RMs; *n* = 6 uninfected, *n* = 6 SIV‐infected). Data were measured by flow cytometry. unpaired t‐test was used to compare percentages of CCR5, CXCR4, and CD62L between SIV‐ and SIV+ animals and between infected SMs and RMs. (D) Correlation between CD62L and age. Spearman correlation was used. The coefficient of correlation and the *p* value (two‐tailed) are shown.
**Figure S2:** Differential monocyte activation signatures distinguish SIV‐infected sooty mangabeys and rhesus macaques. (A) Heatmap analysis of circulating monocyte phenotypes and activation markers in SIV‐infected sooty mangabeys (*n* = 12) and SIV‐infected rhesus macaques (*n* = 3). Data included monocyte subset frequencies (CD14+ and CD14+CD16+) and expression of activation, adhesion, and trafficking markers (CD11b, CCR2, CCR5, CCR8, CD163, HLA‐DR, CD86, Mac387, CD68, CD44v6, and CD4), as well as plasma sCD163 and viral load. Data were analyzed in R using hierarchical clustering and visualized as a heatmap. (B) PLS‐DA and sPLS‐DA analyses (mixOmics R package) performed to assess separation between SIV‐infected sooty mangabeys and rhesus macaques based on monocyte phenotypic profiles. (C, D) Random Forest classification (randomForest package, ntree = 5 000) used to rank variable importance and identify key monocyte‐associated markers contributing to group discrimination. (E) Box plot of CD14‐CD16 + CCR8+ in SIV‐infected sooty mangabeys (*n* = 12) and SIV‐infected rhesus macaques (*n* = 3).

## Data Availability

The data that support the findings of this study are available from the corresponding author upon reasonable request.
