# Supplementary material for: Nonpathogenic SIV Infection in Sooty Mangabeys Is Characterized by a Lack of Activation and Expansion of Monocytes
Source: J Med Primatol. 2026 Aug 13;55(5):e70104. doi: 10.1111/jmp.70104 (PMC13473726; doi:10.1111/jmp.70104)
Supplement: Supplementary file 1 [file JMP-55-e70104-s001.pdf]

Figure S1

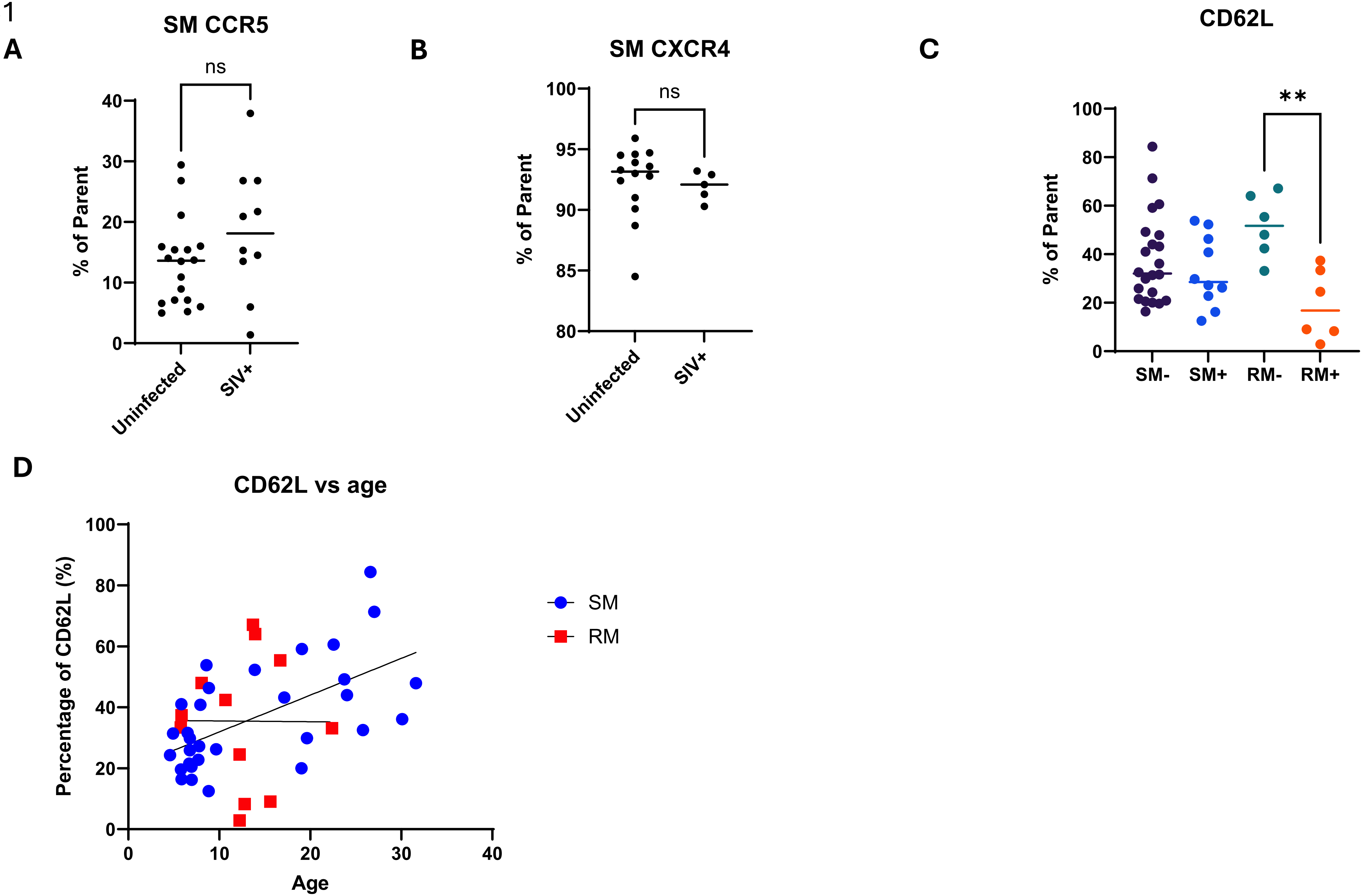
A
SM CCR5
ns
% of Parent
0
10
20
30
40
Uninfected
SIV+
B
SM CXCR4
ns
% of Parent
80
85
90
95
100
Uninfected
SIV+
C
CD62L
**
% of Parent
0
20
40
60
80
100
SM-
SM+
RM-
RM+
D
CD62L vs age
Percentage of CD62L (%)
0
20
40
60
80
100
0
10
20
30
40
Age
SM
RM

Figure S2

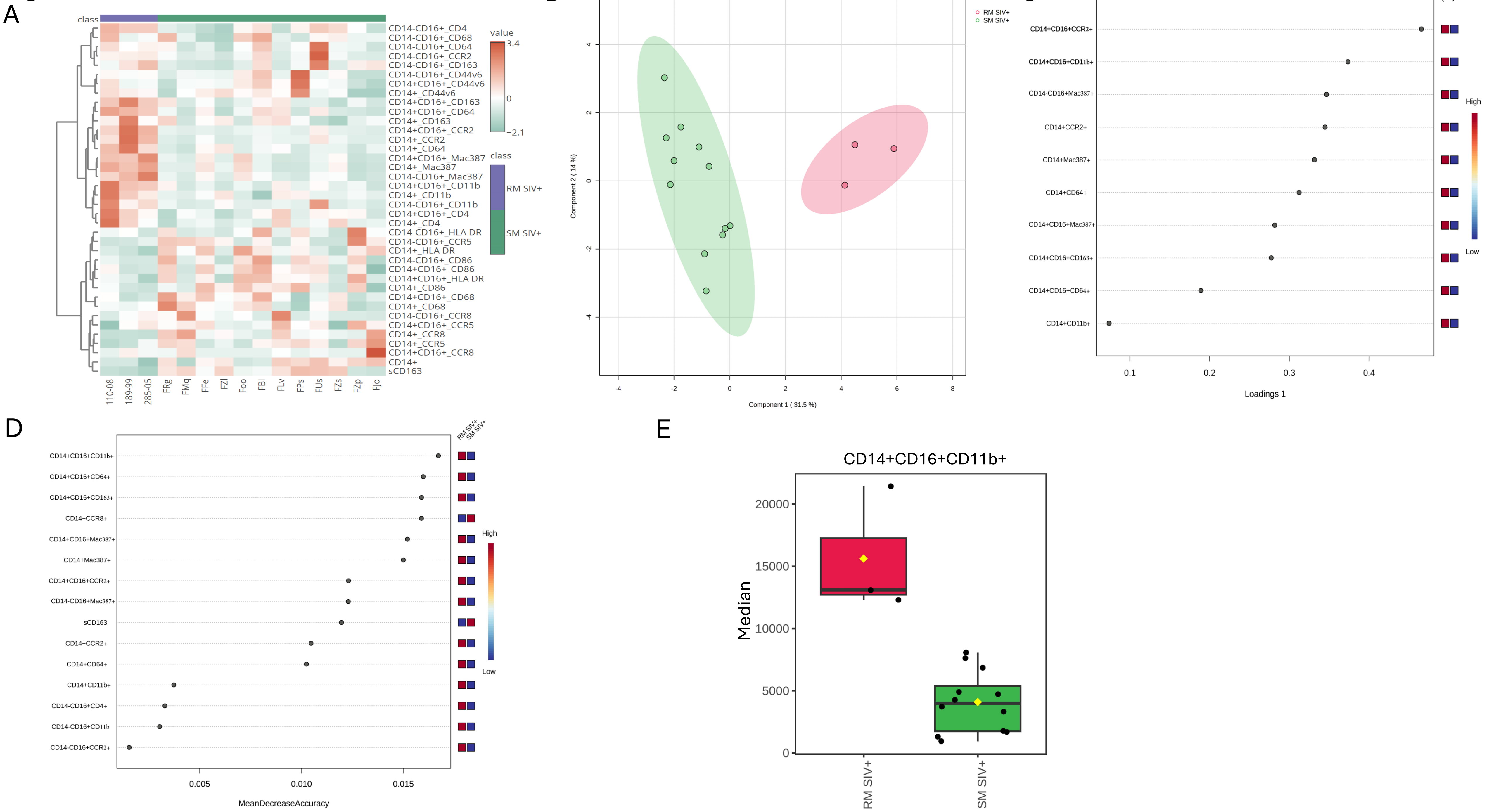
A
class
value
3.4
0
−2.1
RM SIV+
SM SIV+
CD14-CD16+_CD4
CD14-CD16+_CD68
CD14-CD16+_CD64
CD14-CD16+_CCR2
CD14-CD16+_CD163
CD14-CD16+_CD44v6
CD14+CD16+_CD44v6
CD14+_CD44v6
CD14+CD16+_CD163
CD14+CD16+_CD64
CD14+_CD163
CD14+CD16+_CCR2
CD14+_CCR2
CD14+_CD64
CD14+CD16+_Mac387
CD14+_Mac387
CD14-CD16+_Mac387
CD14+CD16+_CD11b
CD14+_CD11b
CD14-CD16+_CD11b
CD14+CD16+_CD4
CD14+_CD4
CD14-CD16+_HLA DR
CD14-CD16+_CCR5
CD14+_HLA DR
CD14-CD16+_CD86
CD14+CD16+_CD86
CD14+CD16+_HLA DR
CD14+_CD86
CD14+CD16+_CD68
CD14+_CD68
CD14-CD16+_CCR8
CD14+CD16+_CCR5
CD14+_CCR8
CD14+_CCR5
CD14+CD16+_CCR8
CD14+
sCD163
110-08
189-99
285-05
FRg
FMq
FFe
FZl
Foo
FBl
FLv
FPs
FUs
FZs
FZp
FJo
B
RM SIV+
SM SIV+
Component 1 (31.5 %)
Component 2 (14 %)
C
RM SIV+
SM SIV+
CD14+CD16+CCR2+
CD14+CD16+CD11b+
CD14-CD16+Mac387+
CD14+CCR2+
CD14+Mac387+
CD14+CD64+
CD14+CD16+Mac387+
CD14+CD16+CD163+
CD14+CD16+CD64+
CD14+CD11b+
High
Low
Loadings 1
D
RM SIV+
SM SIV+
CD14+CD16+CD11b+
CD14+CD16+CD64+
CD14+CD16+CD163+
CD14+CCR8+
CD14+CD16+Mac387+
CD14+Mac387+
CD14+CD16+CCR2+
CD14-CD16+Mac387+
sCD163
CD14+CCR2+
CD14+CD64+
CD14+CD11b+
CD14-CD16+CD4+
CD14-CD16+CD11b
CD14-CD16+CCR2+
High
Low
MeanDecreaseAccuracy
E
CD14+CD16+CD11b+
Median
RM SIV+
SM SIV+
